# Supplementary material for: Specialized RNA decay fine-tunes monogenic antigen expression in Trypanosoma brucei
Source: Nat Microbiol. 2026 Mar 30;11(4):1080–99. doi: 10.1038/s41564-026-02289-4 (PMC13056571; doi:10.1038/s41564-026-02289-4)
Supplement: Supplementary file 1 — Reporting Summary [file 41564_2026_2289_MOESM1_ESM.pdf]

nature portfolio

Corresponding author(s): Joana Faria

Last updated by author(s): Feb 8, 2026

Reporting Summary

Nature Portfolio wishes to improve the reproducibility of the work that we publish. This form provides structure for consistency and transparency in reporting. For further information on Nature Portfolio policies, see our Editorial Policies and the Editorial Policy Checklist.

Statistics

For all statistical analyses, confirm that the following items are present in the figure legend, table legend, main text, or Methods section.

n/a	Confirmed	
☐	☒	The exact sample size (*n*) for each experimental group/condition, given as a discrete number and unit of measurement
☐	☒	A statement on whether measurements were taken from distinct samples or whether the same sample was measured repeatedly
☐	☒	The statistical test(s) used AND whether they are one- or two-sided<br>*Only common tests should be described solely by name; describe more complex techniques in the Methods section.*
☒	☐	A description of all covariates tested
☐	☒	A description of any assumptions or corrections, such as tests of normality and adjustment for multiple comparisons
☐	☒	A full description of the statistical parameters including central tendency (e.g. means) or other basic estimates (e.g. regression coefficient) AND variation (e.g. standard deviation) or associated estimates of uncertainty (e.g. confidence intervals)
☐	☒	For null hypothesis testing, the test statistic (e.g. *F*, *t*, *r*) with confidence intervals, effect sizes, degrees of freedom and *P* value noted<br>*Give P values as exact values whenever suitable.*
☒	☐	For Bayesian analysis, information on the choice of priors and Markov chain Monte Carlo settings
☒	☐	For hierarchical and complex designs, identification of the appropriate level for tests and full reporting of outcomes
☒	☐	Estimates of effect sizes (e.g. Cohen's *d*, Pearson's *r*), indicating how they were calculated

Our web collection on statistics for biologists contains articles on many of the points above.

Software and code

Policy information about availability of computer code

Data collection

Software used for data collection (Immunofluorescence analysis):
Zeiss ZEN and ZEN PRO software v / https://www.zeiss.com/microscopy/int/products/microscope-software/zen.html

Data analysis

SOFTWARE / VERSION / Citation
Bowtie2 / v2.5.4 / Langmead & Salzberg 2012 (doi: 10.1038/nmeth.1923)
Samtools / v1.21 / Li et al 2009 (doi: 10.1093/bioinformatics/btp352)
deeptools2 / v3.5.6 / Ramirez et al 2016 (doi: 10.1093/nar/gkw257)
Artemis / v18.2.0 / Rutherford et al 2000 (https://doi.org/10.1093/bioinformatics/16.10.944)
RStudio / v4.3.1 / Available at https://github.com/rstudio/rstudio
IGV / v 2.18.2 / Robinson et al, 2011 https://igv.org/
Bedtools / v2.31.0 / Quinlan and Hall, 2010 / http://bedtools.readthedocs.io/en/latest/
Picard tools / v3.4.0 / https://broadinstitute.github.io/picard/
Progenesis QI / v2.2 / https://www.nonlinear.com/progenesis/qi/
Mascot Daemon / v2.6.1 / https://www.matrixscience.com/daemon.html
Graphpad / v10.0 / https://www.graphpad.com/scientific-software/prism/
Fiji / v2.9.0 / Schindelin et al 2012 (doi: 10.1038/nmeth.2019)
ThermoFisher Connect Platform
AlphaFold2 / https://alphafold.ebi.ac.uk/
FoldSeek / v10 / https://search.foldseek.com/search
ChimeraX / v1.9 / https://www.cgl.ucsf.edu/chimerax/
InkScape / v1.3 / https://inkscape.org

BioRender
Cytoscape / v3.10.3 / https://cytoscape.org/
Revigo / v1.8.1
FragPipe-Analyst / http://fragpipe-analyst.nesvilab.org

Custom code:
All custom scripts are publicly available at Zendodo (https://doi.org/10.5281/zenodo.15357090).

For manuscripts utilizing custom algorithms or software that are central to the research but not yet described in published literature, software must be made available to editors and reviewers. We strongly encourage code deposition in a community repository (e.g. GitHub). See the Nature Portfolio guidelines for submitting code & software for further information.

Data

Policy information about availability of data

All manuscripts must include a data availability statement. This statement should provide the following information, where applicable:

- Accession codes, unique identifiers, or web links for publicly available datasets
- A description of any restrictions on data availability
- For clinical datasets or third party data, please ensure that the statement adheres to our policy

Mass spectrometry data sets and associated results files are referenced in ProteomeXchange (PXD063534) and are available to download from MassIVE (MSV000097776) [doi:10.25345/C5JS9HM41], currently password protected: username and password provided in the manuscript. Data will be fully available to the public upon publication.
RNA sequencing datasets have been deposited in the European Nucleotide Archive (ENA) under primary accession number PRJEB89423. Processed data and results are available as supplementary data.

Research involving human participants, their data, or biological material

Policy information about studies with human participants or human data. See also policy information about sex, gender (identity/presentation), and sexual orientation and race, ethnicity and racism.

Reporting on sex and gender	N/A
Reporting on race, ethnicity, or other socially relevant groupings	N/A
Population characteristics	N/A
Recruitment	N/A
Ethics oversight	N/A

Note that full information on the approval of the study protocol must also be provided in the manuscript.

Field-specific reporting

Please select the one below that is the best fit for your research. If you are not sure, read the appropriate sections before making your selection.

☒ Life sciences ☐ Behavioural & social sciences ☐ Ecological, evolutionary & environmental sciences

For a reference copy of the document with all sections, see nature.com/documents/nr-reporting-summary-flat.pdf

Life sciences study design

All studies must disclose on these points even when the disclosure is negative.

Sample size	Sample size was not statistically predetermined for the individual experiments. The sample size is appropriate as we were able to robustly detect differences as low as % (versus control) for different biological replicates and independent experiments.
Data exclusions	N/A
Replication	All attempts of replication were successfull Mass spectrometry analysis following proximity labelling experiments was performed using 4 or 6 technical replicates. RNAseq experiments were performed using two to five biological replicates for each cell line. For microscopy analysis, at least 2 biological replicates were used and typically >100 cells were analysed per condition. For RT-qPCR analysis, at least 2 biological replicates were used per cell line; technical triplicates were used for each individual condition and were averaged.
Randomization	No randomisation applied.

Blinding

No blinding applied. For RNA-Seq and Proteomics data, blinding was not applicable because data acquisition and analysis were performed using automated algorithms that do not require subjective interpretation. The results are based on quantitative measurements where the investigator's knowledge of the sample group could not influence the raw output. The only analyses where a certain degree of 'subjectivity' would be expected were some of the quantitative microscopic analyses, but we have mitigated for that by having at least two investigators independently looking at the images.

Reporting for specific materials, systems and methods

We require information from authors about some types of materials, experimental systems and methods used in many studies. Here, indicate whether each material, system or method listed is relevant to your study. If you are not sure if a list item applies to your research, read the appropriate section before selecting a response.

Materials & experimental systems

n/a	Involved in the study	
☐	☒	Antibodies
☐	☒	Eukaryotic cell lines
☒	☐	Palaeontology and archaeology
☒	☐	Animals and other organisms
☒	☐	Clinical data
☒	☐	Dual use research of concern
☒	☐	Plants

Methods

n/a	Involved in the study	
☒	☐	ChIP-seq
☒	☐	Flow cytometry
☒	☐	MRI-based neuroimaging

Antibodies

Antibodies used

ANTIBODY / SOURCE / IDENTIFIER
Mouse anti-Myc 9B11 / New England Biolabs / Cat# 2276S
Mouse anti-Myc 4A6 / Merck-Millipore / Cat# 05-724 RRID:AB_568800
Rabbit polyclonal anti-GFP / Life Technologies (Invitrogen) / Cat# A-6455 RRID:AB_221570
Mouse monoclonal anti-HA / Sigma-Aldrich / Cat# H9658
Mouse anti-EF1α CBP-KK1 / Merck-Millipore / Cat# 05-235 RRID:AB_309663
Mouse anti-Ty / ThermoFisher Scientific / Cat# A-6455 RRID:AB_221570
Rat anti-VSG-2 / Prof. George Cross, Rockefeller University / Hoek and Cross, 1999 (doi.org/10.1006/expr.1998.4369)
Rabbit anti-VSG-2 / Prof. George Cross, Rockefeller University / Hoek and Cross, 1999 (doi.org/10.1006/expr.1998.4369)
Rabbit anti-VSG-6 / Prof. George Cross, Rockefeller University / Hoek and Cross, 1999 (doi.org/10.1006/expr.1998.4369)
Mouse anti-EP procyclin / VWBio-Cedarlene / Cat# CLP001AP RRID:AB_10060662
Rabbit anti-Pol-I / In-house / Glover et al, 2016 (doi: 10.1073/pnas.1600344113)
Rabbit anti-VEX2 / In-house / Faria et al, 2019 (doi: 10.1038/s41467-019-10823-8)
Goat anti-mouse Alexa 488 / ThermoFisher Scientific / Cat# A-11001 RRID:AB_2534069
Goat anti-rabbit Alexa 488 / ThermoFisher Scientific / Cat# A-11034 RRID:AB_2576217
Goat anti-mouse Alexa555+ / ThermoFisher Scientific / Cat# A-32727 RRID:AB_2633276
Goat anti-mouse Alexa 568 / ThermoFisher Scientific / Cat# A-11004 RRID:AB_2534072
Goat anti-rabbit Alexa 568 / ThermoFisher Scientific Cat# A-11011 RRID:AB_143157
Chicken anti-rat Alexa 488 / ThermoFisher Scientific / Cat# A-21470 RRID:AB_2535873
Goat anti-mouse HRP / Biorad / Cat# 1721011 RRID:AB_11125936
Goat anti-rabbit HRP / Biorad / Cat# 1706515 RRID:AB_11125142

Validation

-There was no new antibody specifically generated for this study.

-Antibodies previously generated inhouse:
Rabbit anti-Pol-I (IFA + WB), validation available in Glover et al, 2016 (doi: 10.1073/pnas.1600344113)
Rabbit anti-VEX2 (IFA only), validation available in Faria et al, 2019 (doi: 10.1038/s41467-019-10823-8)

-Antibodies generated by other labs:
Rat anti-VSG-2 (IFA), validation available in Hoek and Cross, 1999 (doi.org/10.1006/expr.1998.4369)
Rabbit anti-VSG-2 (WB), validation available in Hoek and Cross, 1999 (doi.org/10.1006/expr.1998.4369)
Rabbit anti-VSG-6 (IFA & WB), validation available in Hoek and Cross, 1999 (doi.org/10.1006/expr.1998.4369)

-Commercial antibodies (validation available on manufacturer's website):
Mouse anti-Myc 9B11 (IFA) https://www.cellsignal.co.uk/ - Mouse mAb #2276
Mouse anti-Myc 4A6 (WB) https://www.merckmillipore.com/GB/en/product/Anti-Myc-Tag-Antibody-clone-4A6,MM_NF-05-724
Rabbit anti-GFP LifeTechnologies (IFA / WB) https://www.thermofisher.com/antibody/product/GFP-Antibody-Polyclonal/A-11122
Mouse monoclonal anti-HA (IFA / WB) https://www.sigmaaldrich.com/GB/en/product/sigma/h9658?srsltid=AfmBOorjpZabx94q6N6xnQaYergL4UG4cUGG8s14kA4_oJ5ZZ-KC7C7R
Mouse anti-EF1α CBP-KK1 (WB) https://www.merckmillipore.com/GB/en/product/Anti-EF1-Antibody-clone-CBPKK1,MM_NF-05-235?ReferrerURL=https%3A%2F%2Fwww.google.com%2F&bd=1&cid=BIOS-S-EPDF-1148-1107-RC
Mouse anti-EP procyclin (IFA) https://www.biocompare.com/9776-Antibodies/90525-Mouse-AntiParasite-Trypanosoma-bruceiprocyclin-EP-Monoclonal-antibody-Unconjugated-Clone-tbrp1247/

Goat anti-mouse Alexa 488 (IFA) https://www.thermofisher.com/antibody/product/Goat-anti-Mouse-IgG-H-L-Cross-Adsorbed-Secondary-Antibody-Polyclonal/A-11001
Goat anti-rabbit Alexa 488 (IFA) https://www.thermofisher.com/antibody/product/Goat-anti-Rabbit-IgG-H-L-Highly-Cross-Adsorbed-Secondary-Antibody-Polyclonal/A-11034
Goat anti-mouse Alexa555+ (IFA) https://www.thermofisher.com/antibody/product/Goat-anti-Mouse-IgG-H-L-Highly-Cross-Adsorbed-Secondary-Antibody-Polyclonal/A32727
Goat anti-mouse Alexa 568 (IFA) https://www.thermofisher.com/antibody/product/Goat-anti-Mouse-IgG-H-L-Cross-Adsorbed-Secondary-Antibody-Polyclonal/A-11004
Goat anti-rabbit Alexa 568 (IFA) https://www.thermofisher.com/antibody/product/Goat-anti-Rabbit-IgG-H-L-Cross-Adsorbed-Secondary-Antibody-Polyclonal/A-11011
Chicken anti-rat Alexa 488 (IFA) https://www.thermofisher.com/antibody/product/Chicken-anti-Rat-IgG-H-L-Cross-Adsorbed-Secondary-Antibody-Polyclonal/A-21470
Goat anti-mouse HRP (WB) https://www.bio-rad.com/en-jp/sku/1706516-goat-anti-mouse-igg-h-l-hrp-conjugate?ID=1706516
Goat anti-rabbit HRP (WB) https://www.bio-rad.com/en-us/sku/1706515-goat-anti-rabbit-igg-h-l-hrp-conjugate?ID=1706515

Additionally, the antibodies above were tested in Trypanosoma brucei cells or cell extracts using the appropriate controls to ensure signal specificity. For Western-Blot analysis, the antibodies were tested for their ability to generate a single band with the expected molecular weight; in the case of tagged cell lines, a non-tagged control was always included in all experiments. For IFA, in the case of the primary antibodies, these were first tested for their ability to generate a single band with the correct molecular weight following Western-Blot analysis; for tagged cell lines, a non-tagged control was always included in all IFA experiments. With exception of the mouse monoclonal anti-HA, all the antibodies above have been previously used in Faria et al, 2019 (doi: 10.1038/s41467-019-10823-8).

Eukaryotic cell lines

Policy information about cell lines and Sex and Gender in Research

Cell line source(s)

All Trypanosoma brucei brucei Lister 427 cell lines used and generated in this study are described in the methods section. The original Trypanosoma brucei brucei Lister 427 was a kind gift by Prof George Cross (Rockefeller University). The 2T1 cell line (parental line for RNAi and overexpression mutants) was generated in Prof Horn's lab (Alsford et al, 2005; doi:10.1016/j.molbiopara.2005.08.009). The 2T1/T7/Cas9 cell line (parental line for CRISPR/Cas9 precision editing) was generated in Prof Horn's lab (Rico et al, 2018; http://doi.org/10.1038/s41598-018-26303-w). The T7/TetR/Cas9 cell line (parental line for CRISPR/Cas9 endogenous tagging) was generated in Prof Rudenko's lab (Beneke et al, 2017; https://doi.org/10.1098/rsos.170095). The PT1 cell line was a kind gift by Prof David Horn and Dr Sebastian Hutchinson.

Authentication

RNA-seq provided authentication.

Mycoplasma contamination

All parental lines used for subsequent genetic manipulation were Mycoplasma-free by PCR-based detection.

Commonly misidentified lines
(See ICLAC register)

No commonly misidentified cell lines were used.

Plants

Seed stocks

N/A

Novel plant genotypes

N/A

Authentication

N/A
